# Compensatory effects of different exercise durations on non-exercise physical activity, appetite, and energy intake in normal weight and overweight adults

**DOI:** 10.3389/fphys.2022.932846

**Published:** 2022-08-19

**Authors:** Xiao-Mei Liu, Ke Wang, Zheng Zhu, Zhen-Bo Cao

**Affiliations:** ^1^ Shanghai Frontiers Science Research Base of Exercise and Metabolic Health, Shanghai University of Sport, Shanghai, China; ^2^ School of Kinesiology, Shanghai University of Sport, Shanghai, China

**Keywords:** exercise, non-exercise physical activity, appetite, energy intake, behavior compensation

## Abstract

**Objectives:** To examine compensatory changes of different exercise durations on non-exercise physical activity (NEPA), appetite, and energy intake (EI) in normal and overweight adults, and to determine if different body mass index of individuals interact with these compensatory effects.

**Methods:** Ten normal weight adults (nine females and one male; age: 24.0 ± 0.4 years; BMI: 20.7 ± 0.5 kg/m^2^) and ten overweight adults (six females and four males; age: 24.5 ± 0.9 years; BMI: 25.9 ± 0.4 kg/m^2^) participated in this study. The participants completed two exercise trials: short-duration continuous training (SDCT) and long-duration continuous training (LDCT), i.e., a 40 min short-duration and an 80 min long-duration continuous training in a randomized order. Total physical activity and NEPA were monitored using an accelerometer for seven consecutive days, which involved a two-day baseline observation period (C-pre-Ex), three-day exercise intervention period (Ex), and two-day follow-up period (C-post-Ex). Blood samples were collected for appetite-related hormone analysis. Appetite score was assessed using the visual analogue scale. Energy intake was evaluated by weighing the food and recording diaries.

**Results:** The NEPA evaluation showed that it was higher for SDCT than for LDCT in the C-post-Ex period (F (1, 19) = 8.508, *p* = 0.009) in the total sample. Moreover, results also indicated that NEPA was lower for LDCT (F (2, 18) = 6.316, *p* = 0.020) and higher for SDCT (F (2, 18) = 3.889, *p* = 0.026) in the C-post-Ex period than in the C-pre-Ex and Ex periods in overweight group. Acyl-ghrelin revealed a main effect of time in the total sample and in normal weight and overweight groups; it was lower in the C-post-Ex period than in the C-pre-Ex and Ex periods (all *p* < 0.05). Total EI analysis revealed no significant changes in either the total sample or in the normal weight and overweight groups.

**Conclusion:** These findings demonstrate that short duration exercise led to a compensatory increment in NEPA, whereas long duration exercise induced a compensatory decrease in NEPA. Moreover, there was a higher and delayed compensatory response in overweight adults than in normal weight adults. Nevertheless, energy intake was not changed across time, regardless of exercise duration.

## 1 Introduction

Exercise has been universally accepted and used to treat obesity by increasing energy expenditure (Patterson and Levin, 2008). However, in recent years, physiological (Pontzer, 2018) and behavioral (Rosenkilde et al., 2012; Silva et al., 2018) compensations have been detected in response to exercise-induced changes, which has led to conflicting results on weight control by exercise. Of these, some studies observed that an adaptive decrease in non-essential expenditure may occur when exercise interventions result in a negative energy balance, which is called physiological compensation (Pontzer et al., 2016). Additionally, many other studies have found several behavioral compensations, including a decline in non-exercise physical activity (NEPA) (Colley et al., 2010; Schutz et al., 2014; Drenowatz et al., 2015) and increased energy intake (EI) (Stubbs et al., 2002b; Whybrow et al., 2008; Myers et al., 2019), which may minimize or completely offset the exercise-induced increase in energy expenditure. And several studies have suggested that the compensatory phenomenon may associated with fatigue (Stubbs et al., 2002a), psychological factor (Barutcu et al., 2020) or appetite hormonal changes (Ouerghi et al., 2021).

A previous review by Silva et al. (2018) summarized compensatory responses to structured exercise training in adults and suggested that behavioral compensatory variances may be associated with exercise parameters and individual characteristics. Exercise duration (also known as “dose”), which is a key factor, has been the focus of many studies (Stubbs et al., 2002a; Stubbs et al., 2002b; Whybrow et al., 2008). Schutz et al. (2014) investigated the effect of three different durations of walking intervention (30, 60, and 90 min/day) on behavioral compensations in physical activity and found that the compensatory degree after exercise increased progressively along with the exercise duration increased. However, Stubbs et al. (Stubbs et al., 2002a; Stubbs et al., 2002b) and Whybrow et al. (2008) reported that participants who experienced different exercise energy expenditures showed contradictory results in EI compensatory responses and with no NEPA changes. Thus, evidence of the influences of exercise duration on NEPA and energy intake remains inconclusive.

Additionally, interindividual variability, such as body weight, has also been reported to be a controversial issue (Willis et al., 2014). Highlights from the latest article on physiological energy compensation demonstrated that our body might compensate more strongly for the calories burned during activity as we get fatter, which makes losing fat progressively more difficult in overweight and obese individuals (Careau et al., 2021). This prompted us to consider whether the degree of behavioral compensations reacted differently among population with various body weight. However, no previous study has investigated or analyzed the speculation between different populations. Prior studies have largely focused on overweight and obese participants. Thus, knowledge about the effects of exercise on NEPA and EI among normal weight adults is necessary for acquiring novel insights into compensatory differences.

Therefore, the purpose of our study was to determine the behavioral compensations of different exercise durations on NEPA, appetite, and EI in normal and overweight adults, and examine if different body mass indices of individuals interact with these compensatory effects. And we hypothesized that longer exercise duration and higher body weight would induce more decreases in the non-exercise physical activity and more increases in the energy intake after exercise interventions.

## 2 Materials and methods

### 2.1 Participants

Participants were recruited *via* social platforms and face-to-face contact at university campuses in Shanghai. The inclusion criteria were 1) age 18–35 years; 2) body mass index >18.5 kg/m^2^ and <28 kg/m^2^; 3) stable weight (±2 kg) for the past 3 months and without dieting; 4) healthy and not having any diseases known to influence exercise or hormones; and 5) no regular exercise during the 6 months before the study (<150 min/week of moderate-intensity or <75 min/week of vigorous-intensity physical activity, which was evaluated by filling the International Physical Activity Questionnaire before enrollment in the study). A total of 20 participants were included in the study, which consisted of ten normal weight adults [9 females and 1 male, 18.5 ≤ BMI ≤ 23.9 kg/m^2^, according to the Chinese BMI classification reference (Chen and Lu, 2004)] and ten overweight adults (6 females and 4 males, 24 ≤ BMI ≤ 28 kg/m^2^). Written informed consent was obtained from all the participants before the study. The study was approved by the Ethics Committee of Shanghai University of Sport (102772019RT026) and registered in the Chinese Clinical Trial Registry (ChiCTR1900022912).

### 2.2 Study design

#### 2.2.1 Baseline measurement

One week before the training program, anthropometric parameters, including height, weight (DC-250, TANITA, Japan), and resting metabolic rate (RMR) (COSMED K5, Rome, Italy) were assessed after fasting in the morning. Additionally, the maximum oxygen uptake (VO_2max_) was measured at least 2 h after meals using a modified Bruce protocol on a treadmill (Pulsar 4.0; H/p/Cosmos, Germany) to assess cardiovascular endurance and calculate the exercise load. Gas exchange (breath-by-breath mode) and heart rate (Polar RS400, Finland) were monitored simultaneously. The K5 system was calibrated before each experiment. The calibration process consisted of: 1) room air calibration, 2) flow meter calibration, 3) scrubber calibration, 4) reference gas calibration, and 5) delay calibration. All data were smoothed using 30 s average values with the K5 software, which facilitated subsequent calculations.

#### 2.2.2 Exercise intervention program

**FIGURE 1:**
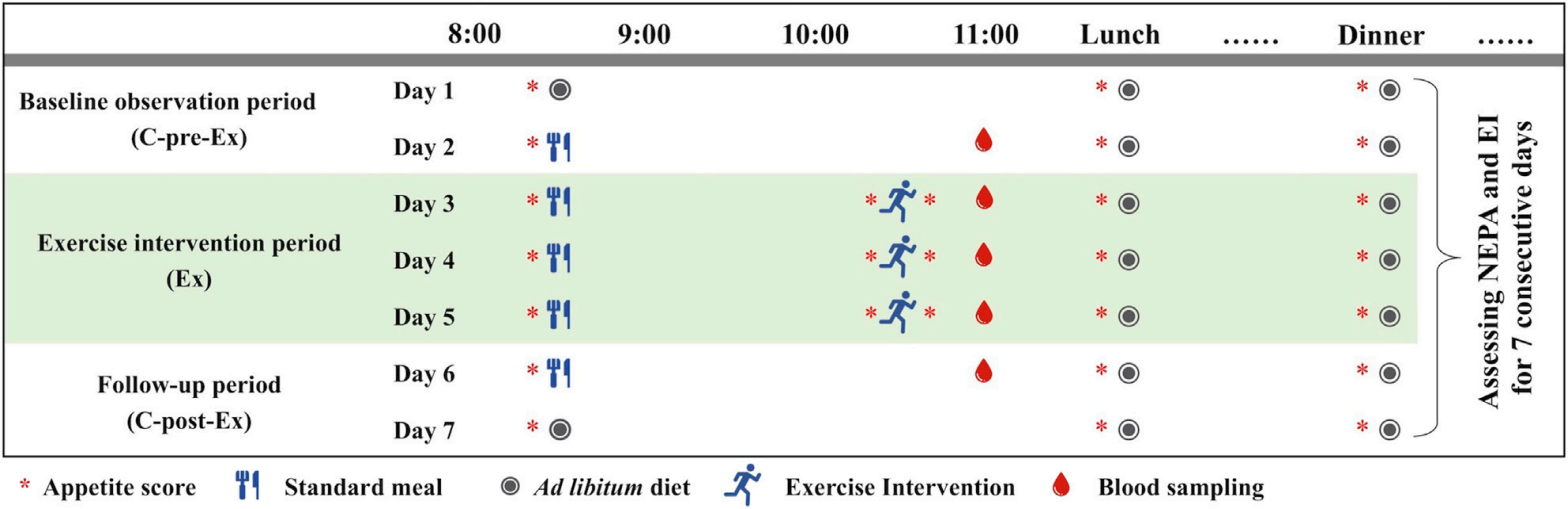
Design of a single experiment.

After the initial measurements, two trials were completed using a randomized crossover design, and the washout period was 7–14 days. Each trial lasted for seven consecutive days, which included a two-day baseline observation period (C-pre-Ex), a 3-day exercise intervention period (Ex), and a two-day follow-up period (C-post-Ex) (Figure 1). The only difference was in the exercise duration of each session during the Ex period. Two experimental conditions were used.

(i) Short-duration continuous training (SDCT), in which participants completed a 40 min running session at an intensity of approximately 65% VO_2max_ in a set time (10:10–10:50) for 3 days. The moderate exercise intensity was determined based on prior relevant studies (Hollowell et al., 2009; Rangan et al., 2011; Bales et al., 2012). And the exercise was performed using a treadmill with the same slope of 5% and calculated speed (Supplementary Table S1).
(ii) Long-duration continuous training (LDCT), during which participants completed two 40 min (double energy expenditure compared to that of SDCT) 65% VO_2max_ running sessions (09:10–09:50 and 10:10–10:50) for 3 days.

All training sessions included a 2-min warm-up and a 2-min cooldown at an intensity corresponding to 50% VO_2max_. Exercise energy expenditure was assessed using the K5 system on the first exercise day. Furthermore, physical activity was measured in both trials *via* accelerometry (GT3X+; ActiGraph, Pensacola, FL, United States) for 7 days. A valid day for adults was defined as ≥10 h. Total physical activity (TPA) was obtained from the accelerometry using established cutoff points (Freedson et al., 1998), which were classified as wear time with counts >100 (all activities were included except sedentary behavior). Non-exercise physical activity was calculated by manually removing the exercise time. Therefore, minutes per hour (min/h) of TPA and NEPA were utilized in the analysis to eliminate the time deduction influences because the C-pre-Ex and C-post-Ex data were not changed. All measurements in females were performed during the follicular phase to avoid hormonal effects on food intake and energy expenditure (Buffenstein et al., 1995). Additionally, the participants had to avoided alcohol consumption and vigorous exercise for ≥24 h before each test.

#### 2.2.3 Blood sampling and biochemical analysis

Five blood collections were performed for all participants on days two, three, four, five, and six during each experiment. Specifically, venous blood samples were collected after the exercise (11:00) per day and at the same time before and after the intervention days (i.e., days two and six). During the preliminary testing, baseline fasting blood samples were drawn into a pre-chilled EDTA-containing vacuum tube and a coagulation-promoting tube. After the collection, the sample in the EDTA tube was immediately centrifuged for 15 min at 4°C and 3,000 rpm, and another tube was leaved at room temperature (22–25°C) for 30 min before centrifugation. The samples were immediately pipetted into the Eppendorf tubes and stored at −80°C until assay. All coding serum baseline samples were sent to an independent laboratory (ADICON Clinical Laboratories, Shanghai, China, INS, accredited by CAP, ISO15189 and CMA) to determine the serum concentrations of glucose (GLU), insulin (INS), non-esterified fatty acids (NEFA), total cholesterol (TC), high-density lipoprotein cholesterol (HDL-C), low-density lipoprotein cholesterol (LDL-C), and triacylglycerol (TG). Additionally, plasma acyl-ghrelin, peptide YY (PYY), glucagon-like peptide 1 (GLP-1), and leptin were measured using ELISA kits (YOYOBIO, China) to assess objective appetite. All the samples from the same participant were analyzed in a same batch. The R-value of the standard linear regression correlation coefficient with the expected concentration was ≥0.990. The intra-assay coefficient of variation was <9%, and the inter-assay coefficient of variation was <11%. Duplicate wells were used and the results were averaged.

#### 2.2.4 Energy intake and appetite

To avoid the influence of food on appetite hormones, breakfast was identical on the five sampling days for the same person in both trials, and they were not allowed to eat any other food until the blood collection was completed. During the rest of the day, food and drinks were consumed *ad libitum*, and a micro digital scale was used to weigh the food intake. Moreover, the participants recorded their food intake and times in a food diary during the intervention days. For the most reliable results, participants were asked to take photos of each meal and send diet records to the researcher *via* WeChat before sleeping every night to avoid forgetting subtle food. Energy intake was calculated according to the China Food Composition, Second Edition (Yang et al., 2009).

Subjective appetite, hunger, and satiety were evaluated immediately by visual analogue scale (VAS) before and after exercise sessions and before each meal. The VAS was composed of four question or scales that provided evaluation of “desire to eat,” “hunger,” “fullness,” and “prospective consumption,” respectively. The average appetite score was calculated using the following formula: appetite score = [desire to eat + hunger + (100 − fullness) + prospective consumption]/4 (Anderson et al., 2002). A higher score indicated better appetite.

### 2.3 Statistical analysis

The sample size was computed *a priori* (G*Power software, v. 3.1.9.3) using an F-test for the analysis of ANOVA with two trial and three repeated measures. We calculated that 15 paired participants would be needed to detect the expected moderate effect size (power = 0.80, f = 0.25, *α* = 0.05) for the non-exercise physical activity changes. Twenty participants were enrolled to allow for potential withdrawals. We recruited normal weight and overweight adults equally to observe the influence of variable body weight factors on compensatory behaviors.

Data were analyzed using SPSS Statistics (version 23.0; IBM Corporation, Armonk, NY, United States). The data analysis was performed in three steps. First, the changes in TPA, NEPA, and EI were compared across 7 days in the total sample with a one-way repeated-measures analysis of covariance (ANCOVA), as was described in a previous similar study (Alahmadi et al., 2011). Second, if there was no effect of time, the TPA, NEPA, EI, appetite score, and related hormones on days 1–2 (C-pre-Ex period), days 3–5 (Ex period), and days 6–7 (C-post-Ex period) were gathered and used for subsequent analyses. The data in the total sample were analyzed using two-way repeated measures ANCOVAs (trial × time), followed by Bonferroni post hoc analyses. Third, we examined whether the changes in physical activity, energy intake, and appetite were equivalent between the normal and overweight groups. We found that these variables were not equivalent. Therefore, body weight, which was a grouping variable, was examined as a potential factor related to the outcomes. Thus, the outcomes in the normal weight and overweight groups were analyzed using a three-way repeated measures ANCOVAs (group × trial × time). In all analyses, sex was included as a covariate to ensure that the statistical balance was not captured by the randomization. All data are expressed as mean ± standard error of the mean (SEM). Statistical significance was set at *p* < 0.05.

## 3 Results

### 3.1 Participants characteristics

The baseline characteristics of participants are showed in Table 1. Overweight participants had higher weight, BMI, RMR, fasting insulin, HOMA-IR, and triglyceride levels, but lower fasting PYY, GLP-1, and leptin levels (all *p* < 0.05).

**TABLE 1:** Participant characteristics.

	Total sample (*n* = 20)	Normal weight (*n* = 10)	Overweight (*n* = 10)
Age (years)	24.3 ± 0.5	24.0 ± 0.4	24.5 ± 0.9
Height (cm)	163.0 ± 1.6	160.9 ± 1.9	165.1 ± 2.4
Weight (kg)	62.2 ± 2.4	53.6 ± 1.8	70.7 ± 2.0*
BMI (kg/m^2^)	23.3 ± 0.7	20.7 ± 0.5	25.9 ± 0.4*
RMR (kcal/24 h)	1,515 ± 68	1,336 ± 56	1,694 ± 96*
Relative VO_2max_ (ml/min/kg)	37.1 ± 1.2	38.4 ± 0.9	35.8 ± 2.2
Fasting glucose (mmol/L)	3.79 ± 0.17	3.58 ± 0.18	4.00 ± 0.30
Fasting insulin (pmol/L)	45.89 ± 7.67	28.25 ± 2.92	63.52 ± 13.07*
NEFA (μmol/L)	409.08 ± 51.97	488.00 ± 72.97	330.16 ± 68.52
HOMA-IR	1.18 ± 0.23	0.67 ± 0.10	1.69 ± 0.40*
Total cholesterol (mmol/L)	2.94 ± 0.20	2.92 ± 0.29	2.96 ± 0.27
Triglyceride (mmol/L)	0.72 ± 0.11	0.46 ± 0.05	0.97 ± 0.18*
HDL cholesterol (mmol/L)	0.85 ± 0.07	0.93 ± 0.09	0.77 ± 0.10
LDL cholesterol (mmol/L)	1.68 ± 0.12	1.61 ± 0.19	1.74 ± 0.17
Fasting acyl-ghrelin (ng/L)	1,624.76 ± 364.23	2,184.52 ± 637.57	1,065.01 ± 289.83
Fasting PYY (ng/L)	210.23 ± 41.84	302.00 ± 63.14	141.84 ± 46.13*
Fasting GLP-1 (pmol/L)	6.73 ± 1.50	9.89 ± 2.52	3.58 ± 0.95*
Fasting leptin (μg/L)	5.84 ± 1.06	7.91 ± 1.86	3.76 ± 0.54*

Data are expressed as the mean ± SEM. *p < 0.05, vs. normal weight group.

### 3.2 Physical activity

The predetermined and actual achieved exercise intensities were in accordance with no difference between the two groups. The energy expenditure of the two trials in overweight adults was significantly higher than that in normal weight adults. Full details are provided in the Supplementary Table S1. In addition, the average wear time of accelerometer was approximately 13 h/day.

Moreover, when comparing the TPA in the total sample across 7 days of testing, a repeated-measures one-way ANCOVA indicated that there was a significant effect of time on SDCT and LDCT (*p* = 0.007 and *p* = 0.000 respectively). No significant differences in either trial was revealed by the post hoc comparisons of the TPA between days 1–2 (C-pre-Ex period), days 3–5 (Ex period), and days 6–7 (C-post-Ex period). Other significant differences between days in different periods are provided in the Supplementary Material. Furthmore, there was no significant effect in NEPA and total EI across 7 days in either the SDCT or LDCT trial. Therefore, averaged data were presented in the subsequent results. Data for the TPA, NEPA, and EI across 7 days for SDCT and LDCT are presented in the Supplementary Table S2.

#### 3.2.1 Total physical activity

**FIGURE 2:**
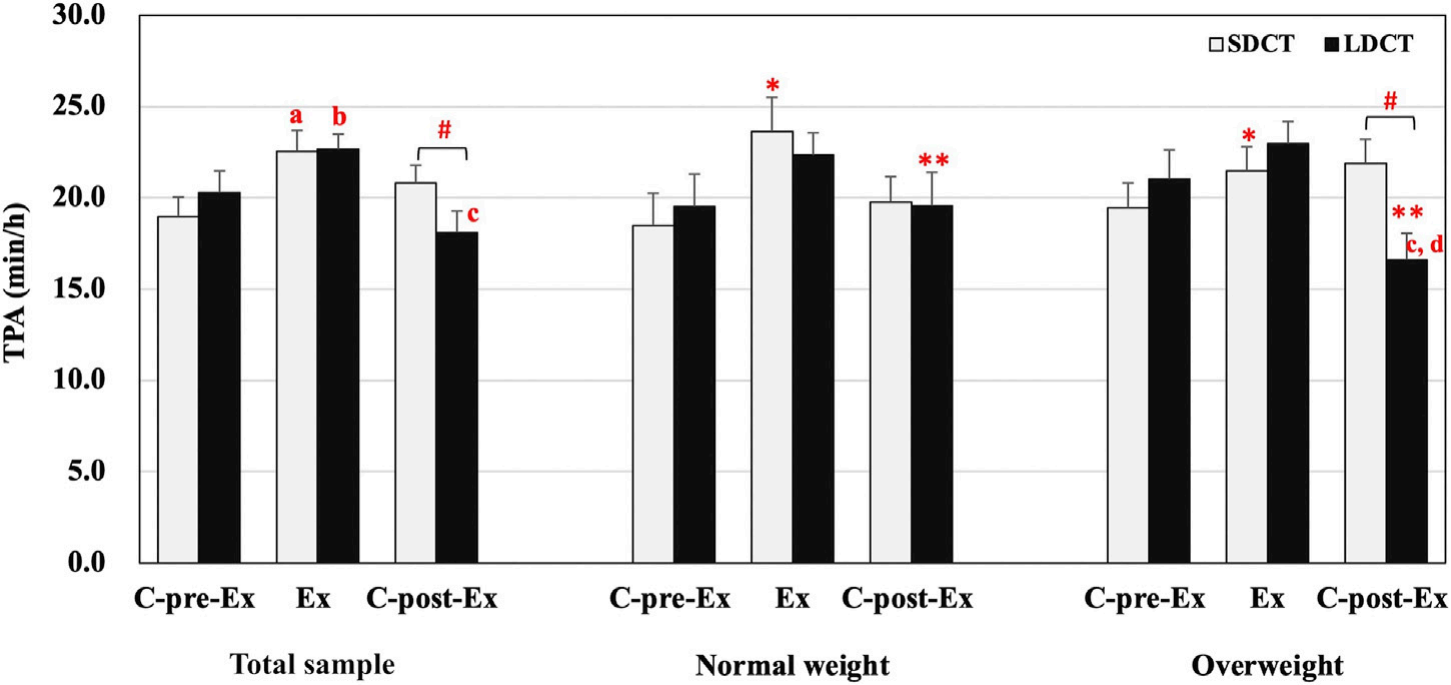
Total physical activity in the total sample, normal and overweight groups. Data are expressed as the mean ± SEM. *Main effect of time for SDCT in the Ex period compared with C-pre-Ex period, *p* < 0.05. **Main effect of time for LDCT in the C-post-Ex period compared with C-pre-Ex and Ex periods, *p* < 0.05. ^#^Significant difference between SDCT and LDCT in the C-post-Ex period, *p* < 0.05. ^a^Significantly different from SDCT in the C-pre-Ex period, *p* < 0.05. ^b^Significantly different from LDCT in the C-pre-Ex period, *p* < 0.05. ^c^Significantly different from LDCT in the Ex period, *p* < 0.05. ^d^Significantly different from LDCT in the C-pre-Ex period, *p* < 0.05. Abbreviations: SEM, standard error of the mean; SDCT, short-duration continuous training; LDCT, long-duration continuous training; C-post-Ex, two-day follow-up period; C-pre-Ex, two-day baseline observation period; Ex, three-day exercise intervention period.

In the total sample, the analysis revealed a significant trial-time interaction (F (2, 38) = 4.272, *p* = 0.021). A post hoc test revealed that the TPA of SDCT was increased in the Ex period compared to that in the C-pre-Ex period (F (2, 38) = 5.710, *p* = 0.011). And the TPA of LDCT was increased in the Ex period compared to that in the C-pre-Ex and decreased in the C-post-Ex period compared to that in the Ex period (F (2, 38) = 11.868, trend *p* = 0.057 and *p* = 0.000, respectively). The post hoc test also indicated that the TPA was significantly decreased in the LDCT trial than in the SDCT trial in the C-post-Ex period (F (1, 19) = 7.825, *p* = 0.011) (Figure 2).

In addition, a three-way group × trial × time interaction was observed for TPA (F (2, 18) = 4.554, *p* = 0.025) (Figure 2). Further, simple two-way interactions were conducted by fixing the levels of one of the interacting factors. Post hoc tests observed a significant trial-time interaction in the overweight group (F (2, 18) = 16.409, *p* = 0.000), a group-trial interaction in the C-post-Ex period (F (1, 9) = 9.595, *p* = 0.013), and main effects of time in SDCT (F (2,18) = 5.645, *p* = 0.012) and LDCT trials (F (2,18) = 16.114, *p* = 0.000). Further analysis exhibited that TPA of LDCT in the C-post-Ex period was significantly decreased compared to that in the C-pre-Ex and Ex periods (F (2,18) = 10.165, *p* = 0.042 and *p* = 0.002, respectively) in the overweight group. And TPA of LDCT in the C-post-Ex period was significantly lower than that of SDCT in the overweight group (F (1, 9) = 19.600, *p* = 0.003). The main effect of time showed that TPA of SDCT was significantly elevated in the Ex period (F (2, 18) = 6.645, *p* = 0.043) compared with the C-pre-Ex period. And TPA of LDCT was significantly decreased in the C-post-Ex period compared with the C-pre-Ex and Ex periods (F (2, 18) = 16.114, *p* = 0.024 and *p* = 0.001, respectively).

#### 3.2.2 Non-exercise physical activity

**FIGURE 3:**
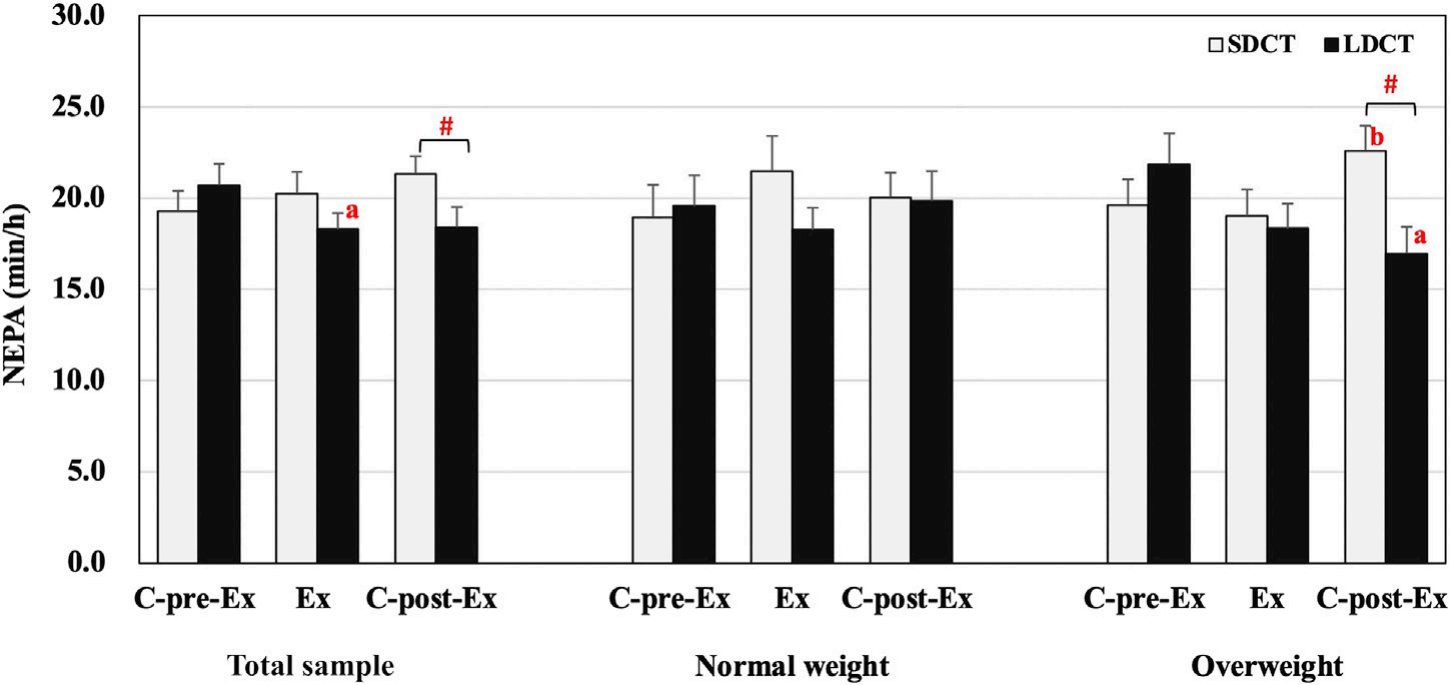
Non-exercise physical activity in the total sample, normal, and overweight groups. Data are expressed as the mean ± SEM. ^#^Significant difference between SDCT and LDCT in the C-post-Ex period, *p* < 0.05. ^a^Significantly different from LDCT in C-pre-Ex period, *p* < 0.05. ^b^Significantly different from SDCT in the Ex period, *p* < 0.05. Abbreviations: SEM, standard error of the mean; SDCT, short-duration continuous training; LDCT, long-duration continuous training; C-post-Ex, two-day follow-up period; C-pre-Ex, two-day baseline observation period; Ex, three-day exercise intervention period.

The analysis showed a significant trial-time interaction (F (2, 38) = 4.871, *p* = 0.013) in the total sample. Further analysis revealed that NEPA of LDCT was decreased in the Ex period compared to that in the C-pre-Ex period (F (2, 38) = 4.415, trend *p* = 0.055). Additionally, NEPA was significantly increased in the SDCT trial than in the LDCT trial in the C-post-Ex period (F (1, 19) = 8.508, *p* = 0.009) (Figure 3). And in a post hoc power calculation, we had 98% (F = 4.0, df = 1) power to detect the 19% variance between SDCT and LDCT trials for the total sample, with an effect size of 0.30.

The three-way repeated measures ANCOVAs showed a group × trial × time interaction for NEPA (F (2, 18) = 5.514, *p* = 0.014) (Figure 3). Post hoc tests revealed a significant trial-time interaction in the overweight group (F (2, 18) = 14.129, *p* = 0.000), a group-trial interaction in the C-post-Ex period (F (1, 9) = 10.160, *p* = 0.011), and a group-time interaction in LDCT trial (F (2, 18) = 4.527, *p* = 0.026). Further pairwise comparisons indicated that NEPA was significantly decreased for the LDCT trial in the C-post-Ex period compared to the C-pre-Ex period (F (2, 18) = 6.316, *p* = 0.020) in the overweight group. Conversely, NEPA in the SDCT trial increased in the C-post-Ex period compared to the Ex period (F (2, 18) = 3.889, *p* = 0.026) in the overweight group. Further analysis also revealed a lower NEPA in the C-post-Ex period for LDCT trial than for the SDCT trial in the overweight group (F (1, 9) = 19.757, *p* = 0.003). Individual NEPA changes during the Ex and C-post-Ex periods are presented in the Supplementary Figures S1,2.

### 3.3 Energy intake

There were no significant main effects of trial, time, or trial-time interaction on daily or lunch energy intake in the total sample. And no significant interactions and main effects were observed for total or lunch energy intake in normal and overweight groups. Full details are provided in Table 2.

**TABLE 2:** Energy intake in the total sample, normal and overweight adults.

	Total sample (*n* = 20)			Normal weight (*n* = 10)			Overweight (*n* = 10)		
	C-pre-Ex	Ex	C-post-Ex	C-pre-Ex	Ex	C-post-Ex	C-pre-Ex	Ex	C-post-Ex
Daily Energy Intake (kcal)									
SDCT	1,686.0 ± 105.8	1,664.4 ± 88.7	1,553.3 ± 108.3	1,582.6 ± 181.2	1,546.5 ± 94.9	1,603.8 ± 151.6	1789.4 ± 109.8	1782.3 ± 145.5	1,502.8 ± 161.3
LDCT	1,635.1 ± 85.0	1713.6 ± 93.5	1,577.5 ± 75.8	1,551.2 ± 98.4	1709.6 ± 125.7	1,560.1 ± 123.4	1718.9 ± 138.7	1717.5 ± 145.2	1,595.0 ± 94.6
Lunch Energy Intake (kcal)									
SDCT	571.7 ± 54.5	479.2 ± 27.3	457.3 ± 50.2	479.3 ± 64.6	428.7 ± 39.9	395.9 ± 62.5	664.1 ± 80.5	529.6 ± 31.6	518.6 ± 76.8
LDCT	524.2 ± 46.9	542.8 ± 35.3	545.6 ± 43.0	534.6 ± 85.3	523.5 ± 46.6	519.4 ± 52.0	513.7 ± 44.6	562.1 ± 54.9	571.7 ± 70.4

Data are expressed as the mean ± SEM. Abbreviations: SEM, standard error of the mean; C-pre-Ex, two-day baseline observation period; Ex, three-day exercise intervention period; C-post-Ex, two-day follow-up period; SDCT, short-duration continuous training; LDCT, long-duration continuous training.

### 3.4 Appetite hormones and score

**FIGURE 4:**
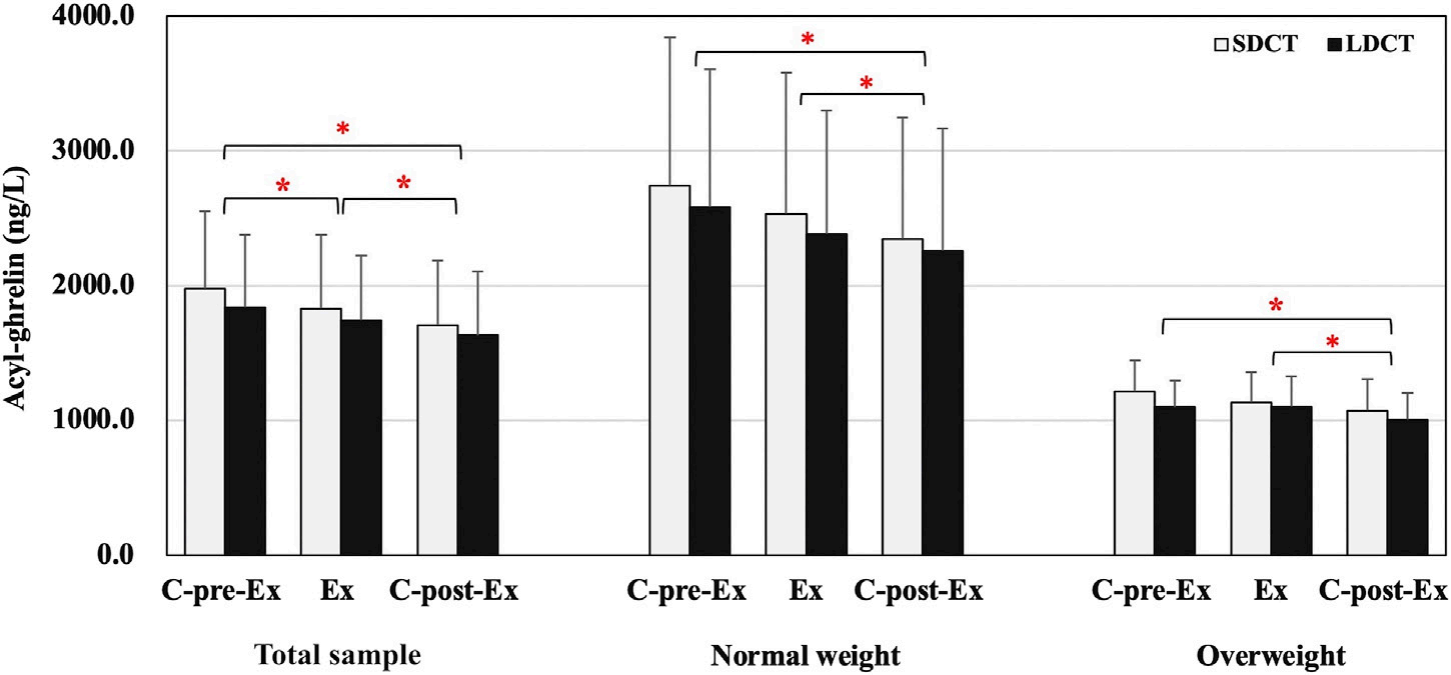
Acyl-ghrelin in the total sample, normal and overweight adults. Data are expressed as the mean ± SEM. *Present the main effect of time, *p* < 0.05. Abbreviation: SEM, standard error of the mean.

In the total sample, the analysis revealed a main effect of time on the acyl-ghrelin levels, which exhibited a significant decrease in the C-post-Ex period compared to that in the C-pre-Ex and Ex periods (F (2, 38) = 0.003, *p* = 0.022 and *p* = 0.003 respectively), and a significant decrease in the Ex period compared to that in the C-pre-Ex period (*p* = 0.000) (Figure 4). Similarly, there was a main effect of time on acyl-ghrelin levels when performing a three-way repeated measures ANCOVAs (F (2, 18) = 8.083, *p* = 0.015). Acyl-ghrelin levels were decreased in the C-post-Ex period than in the C-pre-Ex and Ex periods (*p* = 0.050, trend *p* = 0.071) (Figure 4). Peptide YY, GLP-1, and leptin did not change over the Ex or C-post-Ex periods in the total sample or in normal weight and overweight groups. Full details are provided in the Supplementary Table S3.

**FIGURE 5:**
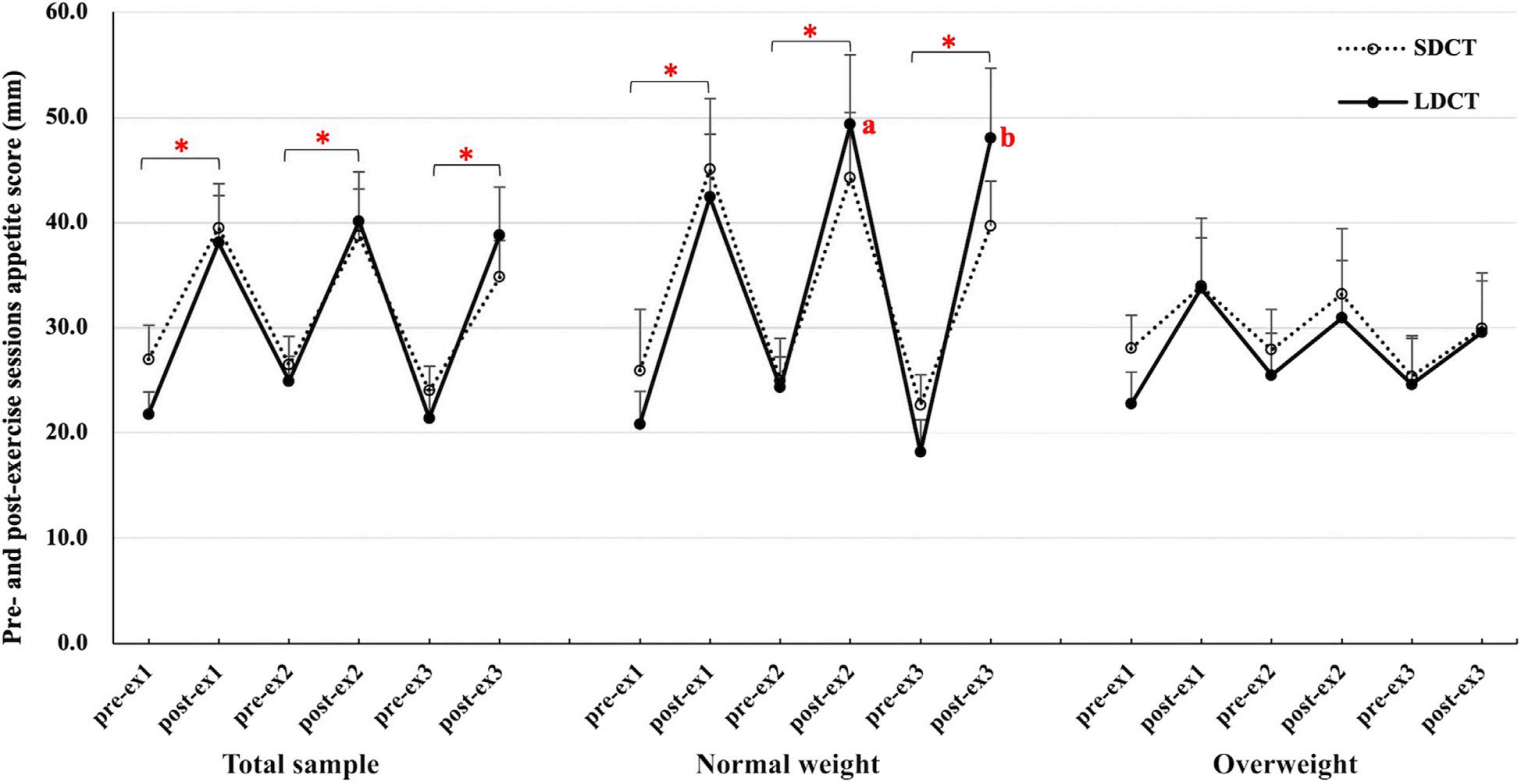
Pre- and post-exercise sessions appetite score in the total sample, normal and overweight adults. Data are expressed as the mean ± SEM. *Significant difference, *p* < 0.05. ^ab^Significantly different from LDCT in the overweight group, *p* < 0.05. Abbreviation: SEM, standard error of the mean.

Furthermore, the pre- and post-exercise appetite scores (evaluated before and immediately after each exercise session) were analyzed during the Ex period (Figure 5). In the total sample, the appetite score was significantly higher after each exercise session than before the exercise session in both trials (all *p* < 0.05). The results of the three-way repeated measures ANCOVAs showed a group-time interaction (F (2.084, 18.758) = 4.472, *p* = 0.025). Post hoc tests revealed a main effect of time in the SDCT trial (F (5, 45) = 6.478, *p* = 0.000) and a group-time interaction in the LDCT trial (F (2.211, 19.899) = 5.320, *p* = 0.012). Further analysis observed that the appetite score was significantly higher after each exercise session than before the exercise session in normal weight group (all *p* < 0.05). And the appetite score of LDCT was higher after the second (F (1, 9) = 4.352, trend *p* = 0.067) and third (F (1, 9) = 5.885, *p* = 0.038) exercise sessions in the normal weight group than that in the overweight group.

Pre-meals appetite score was also analyzed in our study. The results of the pre-breakfast appetite score showed no statistical differences in the total sample. But a main effect of trial was observed for pre-lunch (F (1, 19) = 6.046, *p* = 0.024) and pre-dinner (F (1, 19) = 12.245, *p* = 0.002) appetite score in the total sample, which showed a significantly higher appetite score on LDCT than SDCT. And the results of the three-way repeated measures ANCOVAs were similar with the total sample outcomes. Significant main effects of trial were observed for pre-lunch (F (1, 9) = 12.319, *p* = 0.007) and pre-dinner (F (1, 9) = 13.562, *p* = 0.005) appetite score, which also showed a significantly higher appetite score on LDCT than SDCT. Full details are provided in the Supplementary Table S4.

## 4 Discussion

The purpose of this study was to investigate the short-term influences of different durations of exercise on the NEPA, EI, subjective appetite, and hormones, and observe whether people with diverse body weights reacted differently. The results showed that the NEPA in total sample was decreased after the LDCT trial in the Ex period and increased after the SDCT trial in the C-post-Ex period. Separately, the NEPA was increased after the SDCT trial in normal weight adults, whereas it was decreased after the LDCT trial. In overweight adults, similar compensatory changes for NEPA were observed in the C-post-Ex period, but not in the Ex period. In addition, a decrease in acyl-ghrelin levels indicated that the appetite was suppressed in the total sample and in the normal weight and overweight groups. Simultaneously, the appetite score demonstrated that the appetite was higher after the LDCT trial than after the SDCT trial. However, additional exercise and all appetite changes failed to increase the energy intake.

### 4.1 Total physical activity and NEPA

It can be reasonably concluded from our results and the previous studies (Colley et al., 2010; Manthou et al., 2010; Alahmadi et al., 2011; Schutz et al., 2014; Drenowatz et al., 2015; Herrmann et al., 2015) that compensatory changes in NEPA occur after exercise interventions. The generation of compensatory behaviors has often been identified as a barrier (King et al., 2007) to increasing energy expenditure after exercise in previous studies. However, positive responses, such as an increase in NEPA after exercise, have been observed in several studies. For example, Alahmadi et al. (2011) indicated that a delayed increasing trend in NEPA was observed 48 h after an acute exercise intervention, and Rosenkilde et al. (2012) observed that NEPA numerically increased by 37% in moderate-dose exercise compared with the control group, although the differences were not statistically significant. However, no follow-up studies have explored the cause of this phenomenon. In the present study, we also found that compensatory responses were not necessarily negative. The results showed that the long-duration exercise session resulted in a lower increase in TPA compared with short-duration exercise sessions at the Ex period in normal weight adults. Further analysis revealed that NEPA increased when exercise was of short duration, but decreased after long-duration exercise. This implies that a higher compensation occurred for the long-duration exercise intervention in the Ex period. We considered that the compensatory decline in NEPA after exercise could be partially explained by fatigue due to long-duration exercise. This notion was consistent with Schutz et al. (2014), who found that compensation (%) increased progressively as the duration of exercise increased, and compensation ranged from less than 10% for 30-min of endurance training to 65% for 90-min of training. Therefore, our findings support and further expand the contention that the prolongation of exercise duration is disproportionate to the increase in total physical activity as the 40-min exercise seems more efficient than the 80-min exercise in increasing total physical activity. Thus, further studies are needed to explore more applicable exercise durations that would maximize the effects of exercise.

Additionally, the novel finding is that the degree and emergence time of compensatory responses differed between the normal and overweight groups. In the normal weight group, the TPA significantly increased after both exercise trials in the Ex period and quickly returned to baseline levels in the C-post-Ex period. However, somewhat unexpectedly, in the overweight group, double exercise duration did not accentuate the TPA, and an entirely consistent and non-significant increase in TPA was observed in the Ex period when totally different exercise duration interventions were imposed. Therefore, acute exercise appears to induce a compensatory reduction in NEPA for overweight individuals. Additionally, there were remarkably delayed changes in NEPA after performing exercise interventions, which showed significant increases and decreases in the SDCT and LDCT trials, respectively. These results imply that behavioral compensatory changes in overweight adults were higher than those in normal weight adults because of the non-significant increase in the TPA when different exercise durations were imposed. Moreover, this impact persisted and extended beyond the Ex period, which may be a new discovery and is desirable for weight loss. Further validation is needed in equal sex ratio groups to eliminate the potential impact of the gender, although the sex factor was analyzed as a covariate in this study. Notably, we founded that HOMA-IR was significantly different between normal weight and overweight groups, despite the age and BMIs being within a narrow range. Considering the sedentary inactive participants in our study, this fact may deserve more attention.

### 4.2 Energy intake, appetite, and related hormones

Exercise is thought to potentially modify subsequent appetite and energy intake by influencing hormone secretion (Zouhal et al., 2019). However, the present results revealed no significant compensation or meaningful change in daily EI after the different acute exercise interventions. This finding is in line with some previous acute (Stubbs et al., 2002a; Unick et al., 2010; Balaguera-Cortes et al., 2011; Vatansever-Ozen et al., 2011; Halliday et al., 2021) and chronic studies (Martins et al., 2010; Caudwell et al., 2013; Melzer et al., 2016; Castro et al., 2020), in that EI was almost unchanged after exercise interventions. In an early study by Whybrow et al. (2008) energy intake only began to offset energy expenditure over an intervention during 1–2 weeks; that is, the energy intake not compensated immediately after exercise but changed imperceptibly in the following days. Moreover, it is well known that a subtle diet is extremely difficult to measure accurately, especially under free-living conditions or over a long period. Solving this inherent puzzle remains a fundamental challenge for energy metabolism.

In addition, we assessed whether the changes in post-exercise immediately and pre-meal appetite scores were associated with different exercise programs. Aerobic exercise is generally considered to increase the feeling of hunger when exercise intensity is low (Martins et al., 2010; Caudwell et al., 2013). In the present study, we found that the increase in appetite score immediately after exercise was indeed significant in the normal weight group but not in the overweight group, regardless of exercise duration. The weaker changes in post-exercise appetite scores in the overweight group than in the normal weight group suggest that the sensitivity of appetite perception may be partly decreased. Additionally, the pre-prandial appetite score was analyzed to observe the lagging effect of subjective appetite after exercise. The results showed that the pre-lunch appetite score in the normal weight group and the pre-dinner appetite score in the normal and overweight groups were significantly higher in the LDCT trial than in the SDCT trial, which indicated that the participants considered themselves to be hungrier after the long-duration exercise intervention. This indicates that a longer exercise time or higher energy expenditure would induce more appetite changes, even though compensation for energy intake was not exhibited here.

Numerous studies have evaluated the influences of acute and chronic exercise on ghrelin production. These studies have produced inconsistent results, with an increase, decrease, or no change in circulating ghrelin levels reported in response to acute or chronic exercise (Broom et al., 2007; Ouerghi et al., 2021). In our study, acyl-ghrelin were significantly decreased in the C-post-Ex period than in the C-pre-Ex and Ex periods in the normal weight and overweight groups, with no differences between trials. Consistent with the results of our study, a more recent review indicated that acute exercise suppresses acyl-ghrelin regardless of participant characteristics and exercise, and this influence may persist and extended beyond the end of exercise. Moreover, the review indicated that the effect of ghrelin on appetite and EI seems to be absent or modest (Ouerghi et al., 2021), which may also explain why energy intake was almost unchanged in our study.

In addition, previous studies in lean (King et al., 2011) and obese (Ueda et al., 2009) adults revealed that PYY and GLP-1 levels was increased after exercise. However, inconsistent results have been reported in the literature. A review aimed at examining the changes of appetite hormones after acute exercise in overweight/obese populations observed that acyl-ghrelin was moderately decreased, while.

PYY and GLP-1 were almost not influenced by acute exercise (Douglas et al., 2016). The results of this review appear to be identical with the outcomes of our study, which showed non-significant differences in satiety hormones, regardless of the treatment. It is worth noting that the hormone concentration in the normal weight adults was greater than that in the overweight group in terms of overall level, which reminds us that the exercise-induced changes after chronic exercise in previous research were possibly because of body weight rather than the exercise itself. In many of the studies discussed thus far, it is interesting that there were inconclusive results due to substantial heterogeneity among studies, and responses varied greatly among individuals. However, it is remarkable that EI does not seem to be significantly altered by hormonal alterations (Martins et al., 2015). Our research further validated these findings that the usual diet is a habitual behavior and would not be readily modified immediately after exercise, but rather in an extended period. Therefore, focusing on behavior management may be a more efficient and effective approach to resist compensatory responses among overweight and obese populations.

### 4.3 Advantages and limitations

The specific advantage of the current investigation was that NEPA and energy intake were measured for 7 days, which offered us a chance to monitor behavioral compensations 5 days after the first day exercise. And if the observation period was restricted to 1 day, as designed in a previous study (Jokisch et al., 2012), several compensations of NEPA in the following days would not be detected. Furthermore, we checked behavioral compensations in normal weight and overweight adults to assess whether the alterations in NEPA and EI were associated with body weight, as very few studies have examined the normal weight population. This study has some limitations. In a previous study (Willis et al., 2014), sex was considered an underlying factor, but we did not have an even distribution of genders with 75.0% (15/20) female and 25.0% (5/20) male participants. Thus, sex differences could not be considered simultaneously and it was treated only as a covariate. Secondly, we only concerned on inactive young populations; a larger and more diverse people is needed to verify the findings. Thirdly, the 80-min exercise intervention consisted of two 40-min bouts with a 20-min rest in between rather than one continuous training session. Therefore, behavioral compensatory changes after a continuous 80-min period cannot be verified in our study. Finally, although our sample size was not sufficient in the normal weight and overweight groups, a post hoc power calculation gave us an effect size (across both groups) of 0.04–1.44 and power of 80%–93% for NEPA at the Ex and C-post-Ex periods in both trials.

## 5 Conclusion

The main finding of this study was that exercise duration was related to the behavioral compensations; a short duration exercise led to a compensatory increment in NEPA, whereas long duration exercise induced an apparent compensatory decrease in NEPA. Moreover, we conclude that the degree of compensation is related to body weight, which shows a greater extent of compensatory change in overweight adults. Individuals with higher body weight exhibit a lower sensitivity to physical activity changes through a delayed NEPA compensatory adjustment. Based on the present findings, we propose that a complete exercise program should also pay attention to behavior management after exercise, including maintaining or reducing original energy intake and increasing daily physical activity purposely, which is essential to diminish the probability of behavioral compensations occurrence after exercise.

## Data Availability

The raw data supporting the conclusion of this article will be made available by the authors, without undue reservation.
