# Supplementary material for: Compensatory effects of different exercise durations on non-exercise physical activity, appetite, and energy intake in normal weight and overweight adults
Source: Front Physiol. 2022 Aug 19;13:932846. doi: 10.3389/fphys.2022.932846 (PMC9437276; doi:10.3389/fphys.2022.932846)
Supplement: Supplementary file 1 [file DataSheet1.docx]

Supplementary Material

**Table S1 Actual exercise intensity and energy expenditure**

|  | **Total sample (n=20)** | **Normal weight (n=10)** | **Overweight (n=10)** |
| --- | --- | --- | --- |
| SDCT average VO_2_ (ml/min/kg) | 23.5 ± 0.9 (63%VO_2max_) | 24.3 ± 1.6 (63%VO_2max_) | 22.7 ± 1.0 (63%VO_2max_) |
| LDCT average VO_2_ (ml/min/kg) | 23.3 ± 1.0 (63%VO_2max_) | 22.8 ± 1.5 (59%VO_2max_) | 23.8 ± 1.3 (66%VO_2max_) |
| 50%VO_2max_ treadmill speed (km/h) | 3.8 ± 0.1 | 3.8 ± 0.1 | 3.7 ± 0.2 |
| 65%VO_2max_ treadmill speed (km/h) | 5.2 ± 0.1 | 5.3 ± 0.1 | 5.1 ± 0.2 |
| SDCT energy expenditure (kcal) | 280.4 ± 13.6 | 251.8 ± 17.5 | 309.0 ± 17.2* |
| LDCT energy expenditure (kcal) | 548.0 ± 27.0 | 465.7 ± 27.8 | 630.3 ± 28.3* |

Data are expressed as the mean ± SEM. *p<0.05, vs. normal-weight group.

Abbreviations: SEM, standard error of the mean; SDCT, short-duration continuous training; LDCT, long-duration continuous training

**Table S2 Changes in physical activity and energy intake across 7 days in total sample**

|  | **Day 1** | **Day 2** | **Day 3** | **Day 4** | **Day 5** | **Day 6** | **Day 7** |
| --- | --- | --- | --- | --- | --- | --- | --- |
| **Total physical activity** | | | | | | | |
| SDCT | 19.2 ± 1.1 | 18.7 ± 1.4 | 22.4 ± 1.3 | 22.5 ± 1.7 | 23.7 ± 1.1^a, b^ | 19.9 ± 1.2 | 21.8 ± 1.5 |
| LDCT | 21.5 ± 1.7 | 19.1 ± 1.1 | 21.5 ± 1 | 24.9 ± 1.3^c^ | 22.8 ± 0.7 | 17.8 ± 1.5^d^ | 18.4 ± 1.2^e, f^ |
| **Non-exercise physical activity** | | | | | | | |
| SDCT | 19.6 ± 1.1 | 19 ± 1.4 | 20 ± 1.4 | 20.4 ± 1.8 | 21.5 ± 1.1 | 20.4 ± 1.3 | 22.2 ± 1.6 |
| LDCT | 21.6 ± 1.8 | 19.8 ± 1.3 | 16.7 ± 1.1 | 20.8 ± 1.4 | 17.9 ± 0.7 | 18.5 ± 1.5 | 18.3 ± 1.2 |
| **Total energy intake** | | | | | | | |
| SDCT | 19.6 ± 1.1 | 19 ± 1.4 | 20 ± 1.4 | 20.4 ± 1.8 | 21.5 ± 1.1 | 20.4 ± 1.3 | 22.2 ± 1.6 |
| LDCT | 21.6 ± 1.8 | 19.8 ± 1.3 | 16.7 ± 1.1 | 20.8 ± 1.4 | 17.9 ± 0.7 | 18.5 ± 1.5 | 18.3 ± 1.2 |

Data are expressed as the mean ± SEM.

^a^ *p*<0.05, vs. day 1; ^b^ *p*<0.05, vs. day 2; ^c^ *p*<0.05, vs. day 2; ^d^ *p*<0.05, vs. day 4; ^e^ *p*<0.05, vs. day 4; ^f^ *p*<0.05, vs. day 5.

Abbreviations: SEM, standard error of the mean; SDCT, short-duration continuous training; LDCT, long-duration continuous training

**Table S3 Appetite hormones in total sample, normal weight, and overweight adults**

|  |  | **Total sample (n=20)** | | |  |  | | **Normal weight (n=10)** | | |  |  | **Overweight (n=10)** | | |  |
| --- | --- | --- | --- | --- | --- | --- | --- | --- | --- | --- | --- | --- | --- | --- | --- | --- |
|  | **C-pre-Ex** | | **Ex** | **C-post-Ex** | | | **C-pre-Ex** | | **Ex** | **C-post-Ex** | | **C-pre-Ex** | | **Ex** | **C-post-Ex** | |
| **Acyl-ghrelin (ng/L)** | | | | | | | | | | | | | | | | |
| SDCT | 1977.61 ± 576.14 | | 1830.33 ± 547.5^a^ | 1708.13 ± 477.46^b, c^ | | | 2739.54 ± 1104.34 | | 2528.82 ± 1051.74 | 2346.23 ± 903.71^b, c^ | | 1215.68 ± 230.09 | | 1131.85 ± 225.94 | 1070.03 ± 235.24^b, c^ | |
| LDCT | 1839.74 ± 537.57 | | 1744.11 ± 481.45^a^ | 1632.38 ± 473.34^b, c^ | | | 2579.71 ± 1028.87 | | 2384.64 ± 914.76 | 2260.71 ± 904.42^b, c^ | | 1099.76 ± 199.76 | | 1103.59 ± 225.22 | 1004.05 ± 200.72^b, c^ | |
| **PYY (ng/L)** | | | | | | | | | | | | | | | | |
| SDCT | 162.6 ± 34.31 | | 158.68 ± 36.17 | 146.52 ± 35.68 | | | 198.61 ± 61.37 | | 199.92 ± 65.91 | 187.80 ± 63.87 | | 122.59 ± 22.45 | | 112.86 ± 16.96 | 100.66 ± 21.17 | |
| LDCT | 173.26 ± 36.12 | | 168.34 ± 38.19 | 169.32 ± 41.8 | | | 220.73 ± 68.06 | | 217.06 ± 72.14 | 225.57 ± 78.12 | | 125.80 ± 18.67 | | 119.62 ± 20.04 | 113.06 ± 23.02 | |
| **GLP-1 (pmol/L)** | | | | | | | | | | | | | | | | |
| SDCT | 5.59 ± 1.24 | | 5.38 ± 1.22 | 5.12 ± 1.21 | | | 7.66 ± 2.20 | | 7.22 ± 2.15 | 6.64 ± 2.10 | | 3.53 ± 0.83 | | 3.54 ± 0.93 | 3.60 ± 1.10 | |
| LDCT | 5.66 ± 1.15 | | 5.54 ± 1.12 | 5.3 ± 1.22 | | | 7.68 ± 2.02 | | 7.46 ± 1.93 | 7.18 ± 2.17 | | 3.63 ± 0.75 | | 3.62 ± 0.87 | 3.43 ± 0.90 | |
| **Leptin (μg/L)** | | | | | | | | | | | | | | | | |
| SDCT | 5.38 ± 0.93 | | 5.74 ± 1.18 | 5.73 ± 1.37 | | | 6.61 ± 1.50 | | 7.37 ± 2.07 | 7.70 ± 2.56 | | 4.16 ± 1.04 | | 4.11 ± 1.01 | 3.76 ± 0.73 | |
| LDCT | 5.69 ± 0.86 | | 5.98 ± 1.12 | 6.31 ± 1.42 | | | 6.88 ± 1.37 | | 7.36 ± 2.00 | 8.30 ± 2.68 | | 4.49 ± 0.98 | | 4.59 ± 0.93 | 4.31 ± 0.61 | |

Data are expressed as the mean ± SEM. ^a, b^ *p*<0.05, vs. the C-pre-Ex period. ^c^ *p*<0.05, vs Ex period.

Abbreviations: SEM, standard error of the mean; C-pre-Ex, two-day baseline observation period; Ex, three-day exercise intervention period; C-post-Ex, two-day follow-up period; SDCT, short-duration continuous training; LDCT, long-duration continuous training

**Table S4 Pre-meal appetite score in total sample, normal weight, and overweight adults**

|  |  | **Total sample (n=20)** | | |  | |  | **Normal weight (n=10)** | | | |  |  | **Overweight (n=10)** | | | |  |
| --- | --- | --- | --- | --- | --- | --- | --- | --- | --- | --- | --- | --- | --- | --- | --- | --- | --- | --- |
|  | **C-pre-Ex** | | **Ex** | **C-post-Ex** | | **C-pre-Ex** | | | **Ex** | **C-post-Ex** | **C-pre-Ex** | | | | **Ex** | **C-post-Ex** |  |  |
| **Pre-breakfast appetite score** | | | | | | | | | | | | | | | | |  |  |
| SDCT | 55.80 ± 3.79 | | 57.87 ± 3.62 | 59.94 ± 3.59 | | 64.81 ± 4.14 | | | 62.28 ± 3.07 | 63.71 ± 3.45 | 46.79 ± 5.03 | | | | 53.46 ± 6.46 | 56.16 ± 6.27 |  |  |
| LDCT | 60.71 ± 4.57 | | 64.68 ± 3.27 | 61.47 ± 4.19 | | 71.44 ± 3.17 | | | 68.07 ± 4.24 | 66.26 ± 5.12 | 49.98 ± 7.23 | | | | 60.92 ± 4.99 | 56.68 ± 6.53 |  |  |
| **Pre-lunch appetite score** | | | | | | | | | | | | | | | | |  |  |
| SDCT | 61.85 ± 3.33 | | 63.18 ± 3.07 | 66.28 ± 3.35 | | 64.88 ± 4.82 | | | 65.95 ± 3.16 | 65.79 ± 4.01 | 58.81 ± 4.65 | | | | 60.41 ± 5.30 | 66.76 ± 5.59 |  |  |
| LDCT ^a, b^ | 68.27 ± 3.8 | | 71.69 ± 3.4 | 69.88 ± 3.65 | | 72.15 ± 3.07 | | | 77.33 ± 2.78 | 77.14 ± 2.97 | 64.39 ± 6.95 | | | | 66.04 ± 5.82 | 62.63 ± 5.97 |  |  |
| **Pre-dinner appetite score** | | | | | | | | | | | | | | | | |  |  |
| SDCT | 62.26 ± 2.46 | | 64.71 ± 2.85 | 62.29 ± 3.63 | | 65.01 ± 3.30 | | | 70.48 ± 2.76 | 66.90 ± 4.08 | 59.51 ± 3.61 | | | | 58.95 ± 4.40 | 57.68 ± 5.85 |  |  |
| LDCT ^a, b, c^ | 69.72 ± 3.00 | | 70.01 ± 2.76 | 67.58 ± 3.20 | | 75.51 ± 3.23 | | | 75.82 ± 2.32 | 69.58 ± 3.52 | 63.93 ± 4.50 | | | | 64.19 ± 4.39 | 65.59 ± 5.48 |  |  |

Data are expressed as the mean ± SEM.

^a^ *p*<0.05, a main trial effect in total sample; ^b^ *p*<0.05, a main trial effect in normal weight group; ^c^ *p*<0.05, a main trial effect in overweight group.

vs. the SDCT in C-pre-Ex period in normal weight.

Abbreviations: SEM, standard error of the mean; C-pre-Ex, two-day baseline observation period; Ex, three-day exercise intervention period; C-post-Ex, two-day follow-up period; SDCT, short-duration continuous training; LDCT, long-duration continuous training


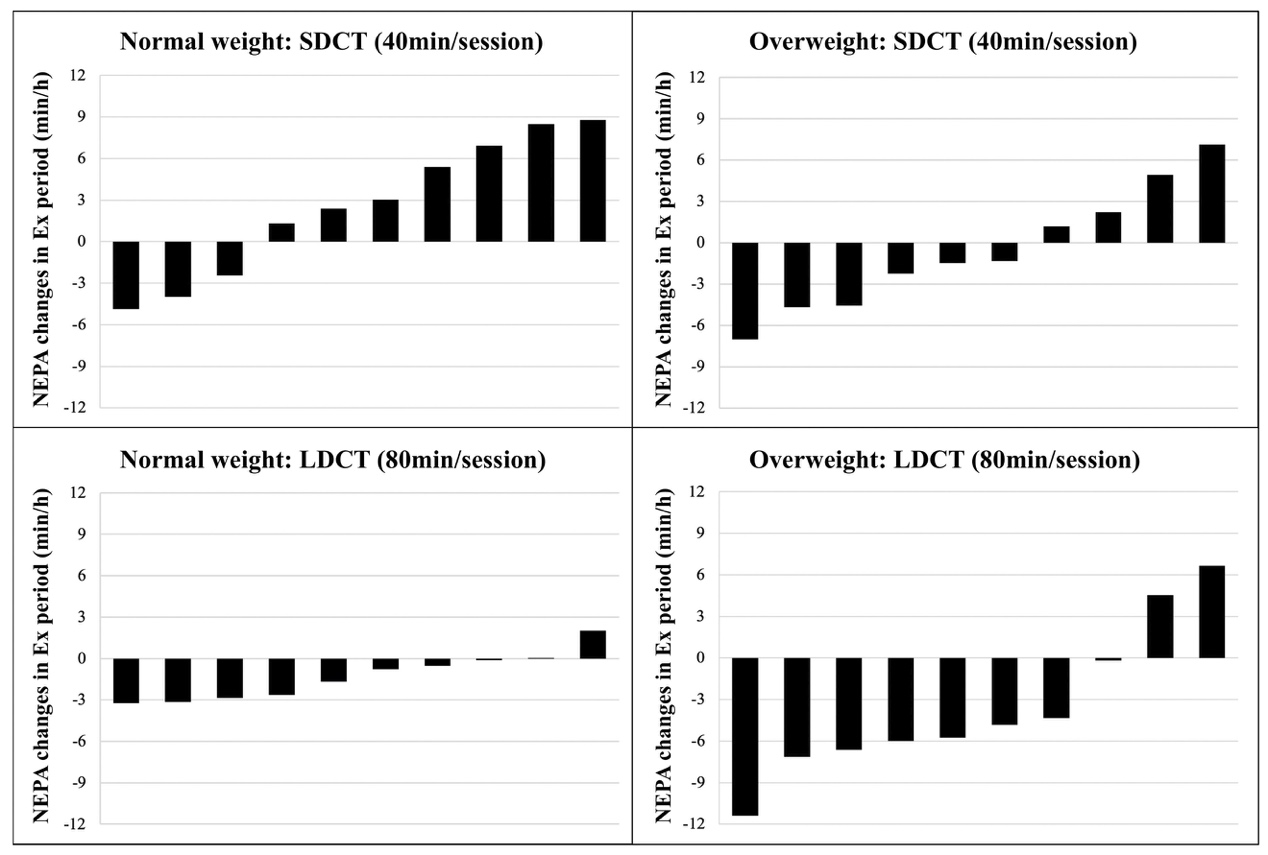


**Figure S1 Individual NEPA changes in Ex period**

The values were calculated using NEPA_Ex_-NEPA_C-pre-Ex_.

Abbreviations: NEPA, non-exercise physical activity; C-pre-Ex, two-day baseline observation period; Ex, three-day exercise intervention period


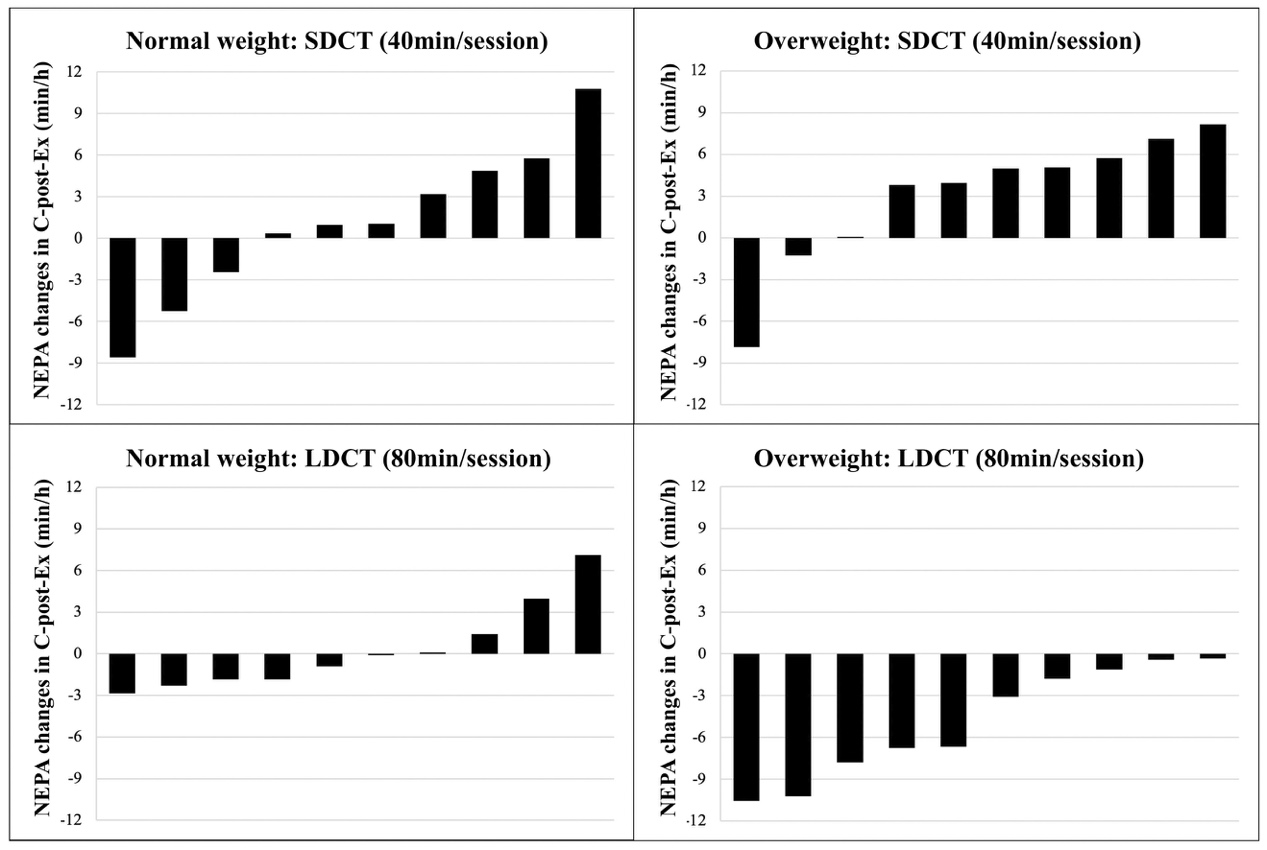


**Figure S2 Individual NEPA changes in C-post-Ex period**

The values were calculated from the NEPA_C-post-Ex_-NEPA_C-pre-Ex_.

Abbreviations: NEPA, non-exercise physical activity; C-post-Ex, two-day follow-up period; C-pre-Ex, two-day baseline observation period
